# Association rare d'une tumeur ovarienne maternelle et d'une tumeur cérébrale néonatale: à propos d'un cas

**DOI:** 10.11604/pamj.2020.35.75.13843

**Published:** 2020-03-16

**Authors:** Houda Rhalem, Houria Knouni, Amina Barkat

**Affiliations:** 1 Service de Médecine et Réanimation Néonatale, HER, CHU Ibn Sina, Rabat, Maroc

**Keywords:** Tumeurs cérébrales fœtales, diagnostic anténatal, métastase placentaire, Fetal brain tumors, antenatal diagnosis, placental metastasis

## Abstract

Les tumeurs cérébrales fœtales sont rares. Le tératome est le type histologique le plus fréquent. Leur évolution est souvent fatale. L'utilisation de l'échographie et de l'imagerie par résonance magnétique permet un diagnostic précoce anténatal. Cependant, le diagnostic définitif repose sur l'histologie. Les métastases placentaires sont rares et semblent compliquées plus souvent les mélanomes malins, les hémopathies, les cancers mammaires et bronchiques. Chez toute femme enceinte ayant présenté récemment un cancer, un examen anatomopathologique du placenta devrait être demandé à la recherche d'un envahissement tumoral. De même, une évaluation néonatale et un suivi pédiatrique prolongé s'imposent. La création de registres des cancers de la femme enceinte est indispensable pour mieux connaître l'épidémiologie de ces cancers ainsi que les facteurs pronostiques maternels et pédiatriques. Nous rapportons un cas de tumeur cérébrale fœtale chez une mère qui a un cancer de l'ovaire récidivant.

## Abstract (en)

Fetal brain tumors are rare. Teratoma is the most common histological type. Their evolution is often fatal. Ultrasound and magnetic resonance imaging allows for early antenatal diagnosis. However, definitive diagnosis is based on histologic examination. Placental metastases are rare and complicate more often the course of malignant melanomas, hemopathies, breast and bronchial cancers. In pregnant women who have recently had a cancer, anatomopathologic examination of the placenta should be requested to detect tumor invasion. Similarly, neonatal assessment and extended pediatric follow-up are required. The establishment of registries collecting all cancer cases diagnosed among pregnant woman is essential for a better understanding of the epidemiology of these cancers as well as of maternal and paediatric prognostic factors. We here report a case of fetal brain tumor in a woman with recurrent ovarian cancer.

## Introduction

Les tumeurs cérébrales fœtales sont rares et constituent 10% des tumeurs fœtales [1]. Elles sont fréquemment diagnostiquées au cours du 3^e^ trimestre, Leur pronostic, généralement mauvais, est fonction de la taille, du type histologique, de la localisation ainsi que du caractère évolutif de la tumeur avec une survie post-natale seulement à 28% [2]. Ces dernières années, l'échographie et l'imagerie par résonance magnétique fœtales, ont permis d'améliorer le dépistage et le diagnostic précoce de ces tumeurs. Même si le diagnostic définitif reste histologique [3]. Nous avons rapporté ici une observation d'une tumeur cérébrale, dont le but est de souligner les difficultés de diagnostic et de prise en charge.

## Patient et observation

**Figure 1:**
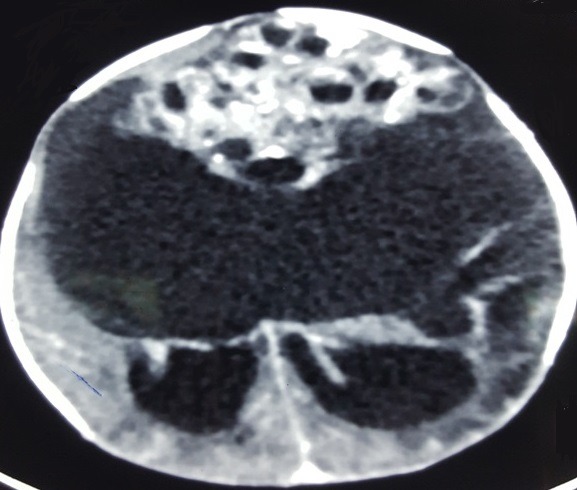
TDM cérébrale (volumineux processus lésionnel à triple composante kystique majoritaire, charnue hétérogène et calcique à l'étage sus tentoriel avec hydrocéphalie)

**Figure 2:**
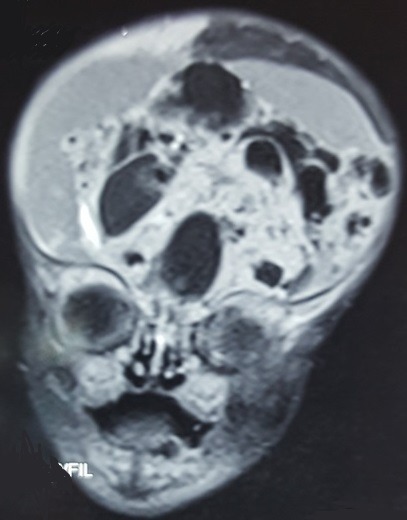
IRM cérébrale (volumineux processus extra axial sus tentoriel qui refoule les hémisphères cérébraux et cérébelleux et responsable d'une hydrocéphalie laminant le parenchyme avec engagement amygdalien)

Nouveau-né de sexe masculin, issu d'une grossesse suivie menée à terme, d'une mère âgée de 23 ans gestité: 4 parité: 2 (2 avortements précoces), suivie depuis 2013 pour tumeur ovarienne gauche avec annexectomie gauche en 2016; notion de consommation du fenugrec pendant la grossesse, sérologies toxoplasmose, rubéole et syphilis (-). Accouchement par voie haute (découverte au cours de la dernière consultation prénatale d'une masse annexielle chez la mère ainsi qu'une masse cérébrale fœtale (diagnostic fait par l'échographie obstétricale complété par la réalisation d'une imagerie par résonance magnétique IRM pelvienne), apgar 10/10, poids de naissance 4400g. L'examen clinique retrouve un nouveau-né rose, tonique, réflexes archaïques présents, fréquence cardiaque à 145 batt/min fréquence respiratoire à 44 C/min tension artérielle 68/42 avec une moyenne de 52, une fontanelle bombante, macrocrânie avec un périmètre crânien à 40 cm. L'examen cardiovasculaire, pleuropulmonaire et abdominal était sans particularités. Pas de malformation cliniquement décelable. Les examens paracliniques comportent: une ponction transfontanellaire qui a ramené 20cc de liquide citrin avec des globules blancs à 3 éléments/mm^3^ et des globules rouges à 30 éléments. Un scanner cérébrale qui a montré la présence à l'étage sus tentoriel d'un volumineux processus lésionnel à triple composante kystique majoritaire, charnue hétérogène et calcique avec hydrocéphalie (aspect évoquant en premier un tératome, cependant un craniopharyngiome ne peut être écarté) (Figure 1). Une IRM cérébrale qui a montré un volumineux processus extra axial sus tentoriel qui refoule les hémisphères cérébraux et cérébelleux et responsable d'une hydrocéphalie laminant le parenchyme avec engagement amygdalien (Figure 2). Les bilans biologiques sont sans particularité. Aucun geste chirurgical n'a été réalisé et le décès est survenu avant toute confirmation histologique.

## Discussion

Les tumeurs fœtales du système nerveux central sont rares. L'incidence des tumeurs cérébrales chez le nouveau-né est de 0,34 par million de naissance vivante et qui représente 0,5 à 1,5% des tumeurs fœtales du système nerveux central [4]. En 1980 Hoff *et al. :* étaient les premiers à décrire un tératome intracrânien diagnostiqué par échographie chez un fœtus au cours de la 28^e^ semaine d'aménorrhée (SA). Depuis lors, d'autres cas de tumeurs cérébrales congénitales ont étés rapportés au cours de la grossesse. Les tératomes et les gliomes sont les plus fréquents [4]. Le diagnostic d'une tumeur cérébrale fœtale repose sur la mise en évidence d'une masse intracrânienne souvent associée à une macrocéphalie (79,2% des cas) et une hydrocéphalie (58,3% des cas). Un hydramnios est fréquemment retrouvé (37,5% des cas).Certaines tumeurs peuvent provoquer une anasarque fœtale par insuffisance cardiaque en cas de shunts artérioveineux [3]. Notre malade présente une macrocéphalie avec hydrocéphalie. La plupart des tumeurs cérébrales fœtales sont supratentorielle. Néanmoins, l'origine est parfois impossible à déterminer [1]. Le recours à L'IRM fréquemment associée à l'échographie 3D et 4D dans la prise en charge anténatale des masses cérébrales. Apparait en post-mortem comme un complément à l'analyse fœtopathologie [5]. Le tératome est la plus fréquente des tumeurs cérébrales fœtales (plus de 62% des cas) [4]. Les tératomes intracrâniens sont le plus souvent diagnostiqués lors des échographies du deuxième ou troisième trimestre, néanmoins un diagnostic précoce bien que rare est possible [4]. Même si le tératome est le plus souvent sus-tentoriel, il peut s'étendre jusqu'aux orbites, l'oropharynx, le nez, le cou ou au-delà de la voute crânienne (17%) [1, 3].

A l'échographie, il s'agit d'une masse solide, volumineuse, complexe, hétérogène (zones tissulaires échogènes, zones hypoéchogènes kystiques, calcifications intratumorales). Les contours sont irréguliers mal définis [1,4,5]. L'IRM retrouve une masse solide multi kystique, hétérogène (multiples zones hypo et hyper intenses en T1 et T2) [3]. Dans notre cas l'aspect évoque en premier lieu un tératome mais le décès est survenu avant toute confirmation histologique. Les astrocytomes (25% des cas) sont de localisation sus-tentorielle, souvent hémisphérique ou thalamique avec un développement unilatéral occupant la majorité, voire la totalité d'un hémisphère cérébrale [6]. Environ 10% des *primitives neuroectodermal tumors (PNET)* ont des contours bien définis et sont modérément vascularisées au Doppler [7]. Les craniopharyngiomes (environ 10%) sont suprasellaires. Ils se présentent comme une masse intracrânienne hyperéchogène, arrondie, centrée sur la ligne médiane et aux contours bien définis. L'étude en Doppler montre une forte vascularisation. L'IRM confirme la localisation suprasellaire et médiane de la tumeur, qui apparait bien limitée [7]. Les papillomes des plexus choroïdes (environ 5%) se développent principalement aux niveaux des ventricules latéraux, du foramen intra-ventriculaire ou des troisièmes et quatrièmes ventricules. A l'échographie il s'agit d'une masse hyperéchogène en « chou-fleur », très vascularisé au Doppler. L'hydrocéphalie est souvent majeure [1,3,8]. Les hémangiomes se manifestent par une masse hyper ou hypoéchogène modérément vascularisée au Doppler couleur et n'ont pas de localisation prédominante. L'IRM retrouve une masse au centre réticulé avec un mélange de signaux d'intensité différente et une bordure hypo-intense. Il existe un hyposignal en T2 [9]. Aucune étude n'a démontré le lien entre la consommation du fenugrec et l'apparition de tumeurs fœtales cérébrales. La croissance des tumeurs est variable en fonction du type histologique mais ce sont les tératomes, les astrocytomes, le craniopharyngiome et les *PNET* qui augment de volume plus rapidement [3].

Dans notre cas, l'antécédent de tumeur ovarienne chez la mère avec notion d'annexectomie gauche et la découverte concomitante d'une masse annexielle au cours de la consultation prénatale fait évoquer une métastase placentaire avec localisation secondaire cérébrale chez le fœtus. L'incidence des cancers associés à la grossesse est estimée à 1/1000 naissances. Les cas d'atteinte fœtale rapportés après un cancer maternel concernent cinq mélanomes malins, trois leucémies, deux lymphomes, deux sarcomes, deux cancers pulmonaires. Parmi ces observations, trois (20%) n'ont pas été confirmées par l'histologie. L'examen macroscopique du placenta peut montrer la présence de nodules de taille variable, pigmentés et rarement achromiques en cas de mélanome malin, blanchâtres dans les autres types de cancer. Le placenta peut être apparemment normal à la coupe. En microscopie optique, l'envahissement de la chambre intervilleuse par des cellules tumorales est l'aspect le plus fréquent [10]. Une étude anatomopathologique du placenta nous aurait avancé dans le diagnostic.

## Conclusion

Le pronostic des tumeurs cérébrales fœtales est fonction du type histologique, de la localisation, de la taille et de l'évolutivité de la tumeur. Il faut savoir distinguer les tumeurs potentiellement curables telles que les tumeurs des plexus choroïdes, des tumeurs rapidement fatales telles que le tératome ou les PNET. Les métastases placentaires de cancers maternels sont rares et semblent compliquer plus souvent les mélanomes malins, les hémopathies, les cancers mammaires et bronchiques. Elles se rencontrent au cours de tumeurs agressives ou évoluées où le pronostic maternel est très sombre. L'examen anatomopathologique placentaire devrait être systématique après l'accouchement de toute patiente ayant présenté récemment un cancer.

## Conflits d’intérêts

Les auteurs ne déclarent aucun conflit d'intérêts.
